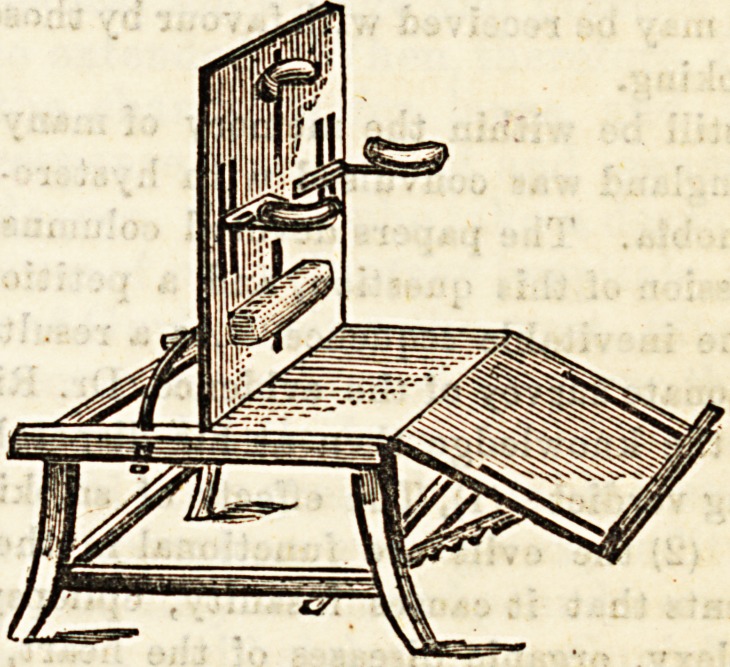# New Drugs, Appliances, and Things Medical

**Published:** 1892-02-20

**Authors:** 


### NEW DRUGS, APPLIANCES, AND THINGS MEDICAL.

[All preparations' appliances, novelties, etc., of which a notice is desired, should be sent for The Editor, to care of The Manager, 140, Strand, London, W.C.]

### SPIKING'S MALT NURSERY BISCUITS.

DOVER STREET, PICCADILLY, &c.

Samples of these old favourites have been sent to us for trial. They are small round cakes, in shape something like "Tops and Bottoms." On boiling they become converted into a thin pulp which will pass easily through the tube of a feeding bottle. We find that in the process of manufacture a large proportion of the starch has been converted into the various sugar forms found after diastatic action further assisted by heat. The resulting material has a high food (carbo-hydrate) value, and is very easily assimilated. As a practical test we chose a case of pneumonia occurring during an attack of influenza in an old person. The biscuits having been well boiled were mixed with hot milk and given through a feeder. The patient liked their taste and digested the mixture perfectly. We have no hesitation in stating that here the biscuits distinctly helped in the treatment of the case. It is of the utmost importance to vary the food in acute cases as well as in the more chronic, because the patient quickly tires of one and the same form of nourishment. We have also used the biscuits recently in the ordinary feeding of infants and children, with gratifying results. We can recommend this firm's manufacture with great confidence.

### LIQUOR CARNIS SUPPOSITORIES.

From the Liquor Carnis Co., Holborn Viaduct, we have received specimens of the above. These are very well made suppositories for rectal feeding, containing two drams of the liquor carnis in the size for adults, and half that quantity in the size for children. We have no doubt that they will be found of great use in those cases where rectal alimentation is made use of, as they are easily introduced, and contain a large amount of readily absorbable food.

### CELERY COFFEE.

THE CELERY COFFEE CO., GLASGOW.

Samples of the above have been submitted to us for examination and report. We have no hesitation in stating that we have found the "coffee" to be a pure article from an analyst's point of view. It is stated to be made from celery alone, and to be particularly useful for dyspeptics and those who suffer from gout or rheumatism. Celery has long enjoyed an anti-rheumatic reputation and from personal and clinical observations, we are inclined to believe this. Apart from these points, however, the manufacturers have succeeded in making a substance, which, when infused, not boiled, yields a very pleasant beverage, which can be drunk with sugar, milk, or cream, as ordinary coffee. It possesses a distinct flavour of its own which is decidedly agreeable. We fancy it has a future before it as a daily beverage. Many people cannot drink either tea, coffee, or cocoa; to them we recommend this as an alternative which, we believe, will be found not only pleasant but satisfying and beneficial to all. Householders should certainly procure some celery coffee without delay.

### AMMONIATED TINCTURE OF QUININE.

J. SELLERS, FARRINGDON ROAD.

We have received a specimen of the above and find it answers the usual tests of the British Pharmacopæia for this preparation. It may, therefore, be used with confidence in the doses usually prescribed, or taken as a prophylactic during the present epidemic period.

### NEWLY INVENTED SPINAL CHAIR.

ALFRED CARTER, HOLBORN VIADUCT.

Mr. Carter has succeeded in inventing a very useful form of chair for the treatment of spinal curvature, which enables the patient to read, write, draw, and play on the piano while reclining in a comfortable position. This does away with a great deal of the tedium and monotony inseparable from chronic spinal complaints. It also counteracts the tendency to stooping so often met with in these cases. The general appearance will be seen from the illustration. The crutches are adjustable vertically and laterally, so as to fit close under the armpits, the neck and waist pillows slide in the centre slot and can be fixed at any point; the back and leg rests are adjustable to any angle; the footboard can be fixed at any required height or angle, and, if required, the back and leg rest can be brought to a level so that the entire length of the apparatus forms a horizontal plane, and then can be used as a reclining board. From the above description it will be seen that there can be few cases, however distorted, which cannot be fitted with an appropriate support in the shape of a spinal chair. We may add that we consider the price as extremely reasonable.

## Figures and Tables

**Figure f1:**